# Fetal heart rate monitoring practices at a public hospital in Northern Uganda – what health workers document, do and say

**DOI:** 10.1080/16549716.2020.1711618

**Published:** 2020-01-20

**Authors:** Elizabeth Ayebare, Wibke Jonas, Grace Ndeezi, Jolly Nankunda, Claudia Hanson, James K. Tumwine, Anna Hjelmstedt

**Affiliations:** a Department of Nursing, School of Health Sciences, College of Health Sciences, Makerere University, Kampala, Uganda; b Department of Women’s and Children’s Health, Karolinska Institutet, Stockholm, Sweden; c Department of Paediatrics and Child Health, School of Medicine, College of Health Sciences, Makerere University, Kampala, Uganda; d Department of Neonatology, Mulago Specialized Women’s & Neonatal Hospital, Kampala, Uganda; e Department of Public Health Sciences, Karolinska Institutet, Stockholm, Sweden; f Department of Disease Control, London School of Hygiene and Tropical Medicine, London, United Kingdom

**Keywords:** Fetal monitoring, fetal heart rate, low resource setting, labor, partograph, intermittent auscultation, health workers, Uganda

## Abstract

**Background**: In Uganda, perinatal mortality is 38 per 1000 pregnancies. One-third of these deaths are due to birth asphyxia. Adequate fetal heart rate (FHR) monitoring during labor may detect birth asphyxia but little is known about monitoring practices in low resource settings.

**Objective**: To explore FHR monitoring practices among health workers at a public hospital in Northern Uganda.

**Methods**: A sequential explanatory mixed methods study was conducted by reviewing 251 maternal records and conducting 11 interviews and two focus group discussions with health workers complemented by observations of 42 women in labor until delivery. Quantitative data were summarized using frequencies and percentages. Content analysis was used for qualitative data.

**Results**: FHR was assessed in 235/251 (93.6%) of records at admission. Health workers documented the FHR at least once in 175/228 (76.8%) of cases during the first stage of labor compared to observed 17/25 (68.0%) cases. Median intervals between FHR monitoring were 30 (IQR 30–120) minutes in patients’ records versus 139 (IQR 87–662) minutes according to observations. Observations suggested no monitoring of FHR during the second stage of labor but records indicated monitoring in 3.2% of cases. Reported barriers to adequate FHR monitoring were inadequate number of staff and monitoring devices, institutional challenges such as few beds, documentation problems and perceived non-compliant women not reporting for repeated checks during the first stage of labor. Health workers demonstrated knowledge of national FHR monitoring guidelines and acknowledged that practice was different.

**Conclusions**: When compared to national and international guidelines, FHR monitoring is sub-optimal in the studied setting. Approximately one in four women was not monitored during the first stage of labor. Barriers to appropriate FHR monitoring included shortage of staff and devices, institutional challenges and mother’s negative attitudes. These barriers need to be addressed in order to reduce neonatal mortality.

## Background

The major causes of neonatal mortality in Low- and Middle-Income Countries (LMIC) are birth asphyxia, complications of prematurity and infections [1]. In Uganda, perinatal mortality is 38 per 1000 pregnancies and 36% of these deaths are due to birth asphyxia [2,3]. Majority of these perinatal deaths could be averted by providing good care such as fetal monitoring during the intrapartum period [4,5]. Appropriate monitoring of fetal wellbeing involves the assessment of the fetal heart rate (FHR), molding of the fetal skull and meconium staining of the amniotic fluid [6].

According to the World Health Organisation (WHO) and International Federation of Gynecology and Obstetrics (FIGO) guidelines, FHR monitoring (FHRM) shall be performed every 15–30 min during the first stage and every 5 min in the second stage of labor [6,7]. In addition, abdominal examination, uterine contractions, fetal movements and maternal pulse shall be monitored. To facilitate recording and guide maternal and fetal monitoring the partograph is recommended. A review of FHRM strategies showed that the use of a partograph to guide monitoring during labor could reduce intrapartum stillbirths [8].

An increasing body of evidence from LMICs suggests that labor monitoring and the use of the partograph is sub-standard [9–11]. Studies from Ghana, Ethiopia, Malawi and Nepal suggest that the FHR was recorded in 25–51% of partographs [12–15]. In rural Western Uganda, a review of partographs showed that FHR was only documented in 2% of the cases [10]. Another study conducted at an urban referral hospital in Uganda found that 62% of partographs had FHR recordings [16]. As FHRM is vital in clinical care, we sought to gain a deeper understanding of FHRM practices by health workers at a public hospital in Northern Uganda. Specifically, we aimed to review birth records with respect to FHRM documentation, observe FHRM in clinical practice, and explore health-care workers’ experiences and perspectives of FHRM.

## Methods

### Study setting

Our study included one maternity unit of a public hospital in Northern Uganda. The maternity unit has 13 beds in the antenatal admission ward, four delivery beds, and 17 beds for post-natal mothers. Sick pregnant women, those in labor, and postnatal mothers are admitted to the unit. On average, there are three midwives during the day, two during the evening and two during the night shift who give care to all women. An obstetrician, a general doctor and two interns provide full-time coverage of the maternity unit. In 2018, 3044 infants were born at the facility. Assisted vaginal deliveries were rarely performed. The cesarean section (CS) rate was 18.6% with around 5.5% performed due to signs of fetal distress. Time from decision to delivery by an emergency CS varied between 30 min and 2 h. In a few cases, it was not possible to perform emergency CS due to lack of supplies and therefore the women had to be transferred to a nearby hospital for the operation. Nearly 6% of newborns were diagnosed with birth asphyxia and the stillbirth rate was 2.2%. There is a special care unit for sick newborns in the facility. When a pregnant woman comes to the unit, she reports at the admission area where a midwife or a doctor assesses and admits her to the antenatal or labor ward depending on her condition. Women in labor are encouraged to ambulate in the corridor or outside the unit. Most women come to the hospital with a companion who may be a relative or friend to help by supporting her during ambulation, providing tea and fetching supplies that may be required by the health workers.

The care provided to women during labor follows the Uganda Clinical Guidelines, which involves the use of a partograph for women who are in active first stage of labor and FHR auscultation for 1 min every 30 min [17]. The Ugandan guidelines are not specific about FHRM during the second stage of labor.

### Study design

We conducted an explanatory sequential mixed methods study [18], which comprised of i) a review of medical records, ii) observation of monitoring practices during labor including FHRM, iii) individual in-depth interviews (IDI) and iv) focus group discussions (FGD) with health workers.

#### Review of medical records

Records of births that took place in January and December 2018 were reviewed by one of the authors (EA), a BSc nurse and a trained research assistant. For conducting the review, we used a predefined checklist that included information on FHR monitoring, plotting of the partograph, vaginal examinations, maternal vital signs and abdominal examination at admission, in the first stage and the second stage of labor (see Table 1). In addition, socio-demographic information of the mothers was collected.

**Table 1.** Record review: FHRM and labor monitoring at admission, during first and second stage

	Admission (n = 251)	First stage (n = 228)	Second stage (n = 251)٭
	%	%	%
Partograph initiated	79.3		
FHRM	93.6	76.8	3.2
≤30 minutes intervals between FHRM		59.0	
>30 minutes intervals between FHRM		41.0	
Contractions	85.3	73.7	
Cervical dilation	94.8	79.3	
State of membranes and amniotic fluid	66.5		
Presenting part	86.1		
Temperature	14.3	9.1	
Pulse	31.9	23.3	
Blood pressure	30.7	22.8	
Urine volume recorded at least once	8.2		
Urine tested at least once	10.8		

٭*only FHR recording at least once reviewed.*

#### Observations

A total of 42 observations of labor monitoring by health workers were carried out between March 2018 and April 2019 by two BSc nurses external to the maternity unit and trained in observation technique. We aimed to make five observations of labor monitoring performed by at least eight health workers involved in intrapartum care as recommended by Stickdorn et al [19]. An observation protocol was developed and used to collect information on FHRM including timing of plotting the partograph, vaginal examinations, maternal vital signs and abdominal examination from admission to birth. The BSc nurses who came daily to the labor ward were engaged only in data collection for the study and *not* involved in the routine care. The health workers were not informed of the exact time when the observations of care would take place to avoid observer effect [20].

#### Analysis of record reviews and observations

Data were entered into SPSS version 23.0 and summarised into frequencies, percentages, means and medians.

#### Individual in-depth interviews and focus group discussions

Individual in-depth interviews (IDIs) were conducted between January and March 2018 and FGDs in January and July 2019. All midwives and medical doctors working in the maternity unit were informed about the study and invited to participate. Those who were willing to participate contacted EA or the research assistants (BSc nurses) to make an appointment for the interview. Eleven health workers participated in the IDIs and seven in two FGDs. The IDIs and FGDs were conducted at the participants’ convenient time in a quiet room at the hospital. The interview guide included questions mainly on FHR monitoring and also about the recording of other parameters in the partograph. The individual interviews lasted for 30–60 min and were performed by one of the authors (EA) and a BSc nurse trained in interview technique. The FGDs took 60 to 90 min and were moderated by EA. The interviews and the FGDs were tape-recorded and transcribed verbatim.

Content analysis was done according to the inductive process described by Elo and Kyngäs [21]. We used the steps described by Erlingsson and Brysiewicz [22] which involve identification of meaning units from transcripts, condensing the meaning units, coding, grouping of codes, categorizing and developing themes. The transcripts were read through several times by EA and AH to get a sense of the whole. Transcripts were then imported to the Atlas ti software for coding. Meaning units were then selected, condensed and coded by EA and reviewed by AH. The codes were discussed by EA, AH and WJ and grouped into categories and themes. Further discussions amongst EA, AH, WJ and the other authors lead to refinement of the final themes. The analysis process is illustrated in Table 2 where one theme, categories, codes and meaning units are presented.

**Table 2.** The process of coding, categorizing and developing one of the themes

Meaning Unit	Condensed unit	Codes	Categories	Theme
*When a mother is in labor we need to assess the fetal heart after every 30 minutes …*	The FHR is monitored every 30 minutes	FHRM interval of 30 minutes	The practice of FHRM	The dilemma of knowing and being able to perform FHRM
*“When the next shift comes we always call the mothers in labor ward so the next shift listens to the fetal heart*	Mothers are called at beginning of every shift for FHRM	FHR monitoring at every shift		
*But for the fetal heart-it should be done half hourly but sometimes sincerely it may not be possible for a mother to be called in for checking every 30 minutes*	FHRM monitoring every 30 minutes is sometimes it’s not possible	Difficult to monitor FHRM as recommended		
*“I would also advocate for close monitoring using a partograph because it can show if anything is wrong. So midwives should do close monitoring not estimating, but close monitoring say of the fetal heart.*	Close monitoring of the FHR should be done using the partograph without estimating	Close monitoring of the FHR. Monitoring should not be estimated		
*… and do general fetal monitoring, its (fetal) movements. The mother can say this child never moved since I came last week or for the last two days, we may feel this one here may have a problem. Then we ask the mother if it is kicking*	General fetal monitoring involves asking the mother is she feels fetal movements and kicking	use of fetal movements to tell fetal wellbeing		
*… also these ones who tell you that ‘musawo’ (health worker) water has come out and once you see the colour has started to change; don’t wait until when it (stops talking) but once you see it is greenish, you sight the meconium +1 you start querying why; by the time it has reached there, there is already a problem”*	when the membranes rupture, and the colour of the amniotic fluid is greenish, there is a problem	Colour of amniotic fluid an indicator of fetal condition		
*‘Actually when you don’t monitor a mother; you yourself when something happens you are psychologically traumatized seriously.’*	Failure to monitor causes psychological trauma	Psychological trauma due to failure to monitor	Emotional consequences of failure to monitor FHR	

### Ethical approval

The study was approved by the School of Health Sciences Research and Ethics Committee of Makarere University (SHSREC). Administrative clearance was obtained from the Hospital’s research and ethics committee. Written informed consent for the in-depth interviews and observation of health workers while performing care was obtained. Anonymity was ensured by using serial numbers and de-identification of the interview transcripts. Permission was sought from the unit heads to conduct observations of care during labor.

## Results

### Review of medical records

**Figure 1.**
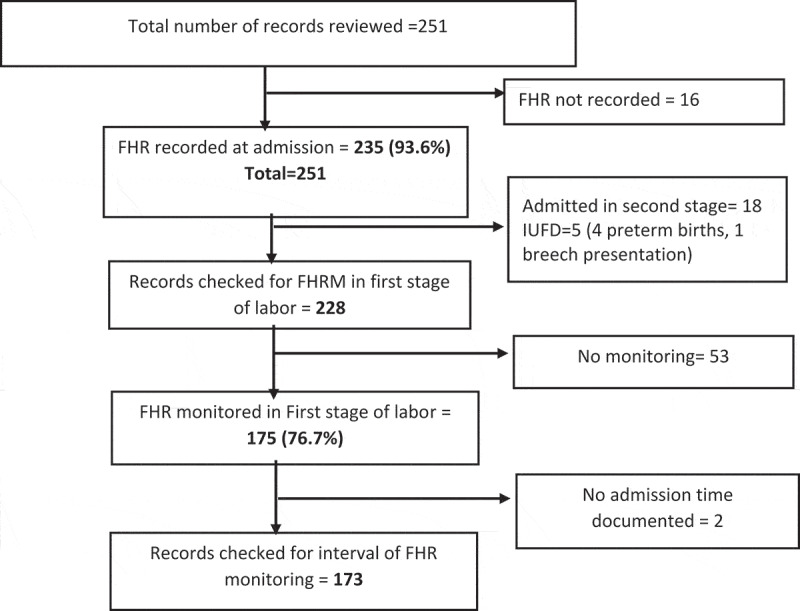
Flow chart for review of medical records

Of the 251 reviewed records, 235 (93.6%, [95% CI: 89.9–96.3]) had FHR recordings at admission. We excluded 23 records of women who were admitted during the second stage of labor and those with intrauterine fetal death (IUFD) to get 228 cases in the first stage of labor. Of these, FHR was monitored at least once during the first stage of labor in 175 (76.8%, [95% CI: 70.7–82.1]) cases. For details see the flow chart (Figure 1). The median interval between FHR measurements was 30 (IQR: 30–120) minutes based on 173 records in which FHR was monitored at least twice within a known time period. Intervals of FHRM during the first stage of labor exceeded 30 min in 41.0% of cases as shown in Table 1.

In the second stage of labor, only 3.2% of the cases were monitored. In a few cases, temperature, pulse, blood pressure, urine measurements and tests were performed. The partograph was used for two-thirds of the cases. Characteristics of the mothers and newborns whose records were reviewed are presented in Appendices A and B.

### Observations

#### Fetal heart rate monitoring

Of the 42 observed births, FHR was checked at admission for 40 (95.2%, [95% CI: 83.8–99.4]) cases. During the first stage, FHRM was observed in 68.0% [95% CI: 46.4–85.0] of the women and in the second stage, monitoring was not observed in any of the cases. Notably; one of the mothers was in the second stage for more than 2 h but not monitored. In a subsample of 27 women, FHRM intervals were noted from admission to birth. The median time interval between FHRM was 139 (IQR: 87–662) minutes. The interval between assessments exceeded 30 minutes for most (96.2%) cases (see Table 3). The device used for FHRM was a fetoscope. Three of the 42 women observed had an emergency cesarean section. Twenty-nine (69.1%) infants were active and/or cried immediately after birth while 11/42 (26.2%) were floppy and needed suctioning and/or ventilation. There were no stillbirths. The partograph was used in 87.8% of the cases and plotting in real time was done in 39.0%. Vaginal and abdominal examinations were performed for most cases (95.2%) at admission. In one-third of the observations, blood pressure was measured (Table 3). The initial assessment of women at admission took approximately 5 (range: 1–10) minutes.

**Table 3.** Observation: FHR and labor monitoring at admission, during the first and second stages

	Admission (n = 42)	First stage (n = 25*)	Second stage (n = 27)
	%	%	%
Partograph used	87.8		
Partograph plotted in real time	39.0		
FHR auscultation performed	95.2	68.0	0
≤30 minutes intervals between FHRM		3.8	
>30 minutes intervals between FHRM		96.2	
Vaginal examination performed	95.2	72.0	
Abdominal examination performed	95.2	56.0	
Blood pressure checked	28.6		
Urine/blood samples collected at least once	2.4		

*Two mothers had very strong contractions and went directly to the delivery bed. One of these gave birth after 2 min and the other woman after 20 min.

### Individual in-depth interviews and focus group discussions

Eighteen midwives and doctors participated in the IDIs and FGDs (see Appendix C for characteristics). Two themes emerged from the data: ‘The dilemma of knowing and not being able to perform FHRM’ and ‘Barriers to adequate FHRM’. The themes and categories with supporting quotes from participants are described below in detail.

## Theme 1: the dilemma of knowing and not being able to perform FHRM

### The practice of FHRM

Most health workers reported that the FHRM should be performed at admission and then every 30 min as specified in the Ugandan guidelines. They also stated that it was difficult to achieve this interval. Instead, often FHR was only monitored at the beginning of every shift and during ward rounds.

*‘When the person on shift hands over the mother to you, you are the one to monitor the fetal heart. In between work, she monitors and when she is about to leave work, she also monitors and hands over to the next person’*. (IDI#11, Midwife)

Close monitoring of FHR was said to be performed when there were signs of fetal distress, labor complications or for women with medical risks like malaria, HIV or urinary tract infections. Health workers employed other mechanisms to examine the status of the fetus such as asking the mother about fetal movements and assessing the color of the amniotic fluid throughout the whole labor.

### Emotional consequences of failure to monitor FHR

Health workers mentioned that the inability to monitor adequately caused them stress, psychological trauma and sleeping difficulties. One health worker described a situation when an intrapartum stillbirth had occurred and the mother had not been monitored according to guidelines. The health worker found this experience too difficult to handle.

*‘Actually, when you don’t monitor a mother; you yourself when something happens you are psychologically traumatized seriously.’* (FGD1, Midwife 1)

## Theme 2: barriers to adequate FHRM

### Shortage of staff as a barrier to FHRM

The health workers acknowledged a difficulty in performing FHRM every 30 min when they were single staff on duty and it was difficult to find time within the busy schedule for appropriate monitoring.

*“Like the contractions you are supposed to take 10 minutes counting them, and here you find like eight mothers coming at the same time and maybe you are the only person here or you are two, so taking 10 minutes to count contractions for effective monitoring of these mothers is a big challenge”*. (IDI#4, Doctor)

In the second stage of labor, FHRM was rarely performed because the health workers conduct the births alone. Midwives explained that they would need to remove the sterile gloves to listen to the FHR which was perceived as cumbersome. Another reason was the mother’s discomfort arising from auscultation of the FHR amidst frequent contractions.

*“Those strong contractions; sometimes you want to listen but the mother is alarming, the abdomen is too tense and you can’t even press to get the fetal heart.”* (FGD2, Midwife 3)

### Shortage of devices to monitor FHR

Fetal heart rate was mainly monitored using a fetoscope but some participants reported that the number of fetoscopes was not enough for the unit. Not all health workers had a watch for counting the FHR and the wall clock was sometimes out of sight.

*“Those ones are there like the fetoscopes are there, the timers; we encourage the midwives to have their watches but this has been a problem to some of them you put your fetoscope and you are looking at the clock up there (meaning the wall clock on one side of the labor ward)”* (IDI#2, Doctor)

Other devices such as Doppler and electronic fetal monitors were sometimes available but the gel was not always available. A knowledge gap in the use of the Doppler and fetal monitors was reported. Accessing the ultrasound scan when health workers needed to confirm fetal heartbeat was a challenge.

*“Like the fetal monitor is supposed to be charged; if it isn’t fully charged like maybe power wasn’t there or maybe someone forgot to put it on charge it will give you a wrong finding. And then also for the Doppler, it uses a KY jelly and sometimes if you find that the jelly isn’t there and you can’t use it. So we resort to the fetoscope which is reliable and doesn’t need anything.”* (FGD2, Midwife 4)

### Barriers to documentation of FHR

Documentation of FHR was mentioned to be a challenge, especially in the latent phase. This is because the FHR is supposed to be documented on the partograph which is usually started when the woman is in active labor (i.e. has a cervical dilatation of 4 cm). Differences in how to plot the partograph were a source of disagreement among the staff. Difficulties were said to arise especially if one staff member took over the care of a mother and had to continue on an already opened partograph. Some health workers therefore resorted to plotting after birth to have a perfectly filled in partograph. Others said that when they monitored the FHR but plotted the findings later, it may have resulted in forgetting the findings and inaccurate recordings.

*“Because we have been trained differently in other schools. So when I start a partograph and I leave it hanging when the patient hasn’t yet delivered, the other one who comes might find it difficult to complete. […]. So I think they should give us one training of how to fill it because sometimes everyone says, no this isn’t like this. That is why sometimes other people don’t even open the partograph. Someone just does it after delivery or begins it a little and leaves it hanging.”* (FGD1, Midwife 2)

A form that was put on the notice board and updated at the beginning of every shift had been introduced in response to previous adverse experiences regarding documentation illustrated by the below citation.

*“Why we did that is because of such instances, a woman lost a precious baby and no one knew about this woman except the mother and her attendant. And to make it worse those days we used to like writing in the antenatal cards, so she wrote all the findings in the antenatal cards, nothing was written in the admission book or clerk form.”* (FGD1, Midwife 3)

### Institutional barriers to FHRM

Shortage of beds was cited to hinder the health workers’ ability to monitor. When the number of women in labor were more than the available beds, it was difficult to listen to the FHR every 30 min.

*“ … like for us there we have only 4 couches (delivery beds) so if you are to come and put mothers on all beds and tie those things (fetal monitors) there again there will be no space. So maybe for those whom you think at least need to be monitored very closely … ”* (FGD2, Midwife 3)

### Women’s perceptions toward FHRM

Health workers expected the women in labor to take responsibility of ensuring that they were monitored according to schedule. However, health workers recounted times when women did not want to be examined frequently. In addition, health workers mentioned that when women were told to ambulate, they sometimes returned home to perform their daily activities only to return to the hospital in the second stage of labor.

*“You admit a mother and she knows very well, you have told her that the cervix is opening just move around here but instead she will go back to town. Patients here, they move up to the market and come back when the baby’s head is out. Actually you haven’t monitored her, you started the partograph and she is nowhere to be seen.”* (FGD1, Midwife 4)

## Discussion

In our study on FHRM practices at a public hospital in Northern Uganda, we used three scientific approaches, including reviews of birth records, observations of clinical practice, individual interviews and focus group discussions.

While FHRM was almost universally assessed at admission, median intervals between FHRM were around 30 min in the records and as high as 139 min according to observations. Of particular note, there was no monitoring of FHR observed during the second stage of labor. Barriers to adequate FHRM included shortage of staff and monitoring devices, institutional challenges, documentation problems and women’s own perceptions towards monitoring. Health workers were very aware of the recommended FHR monitoring guidelines, which implied a delicate dilemma for them: as they know the standards of FHRM but are not able to fulfill those guidelines.

The review of records and observations both indicate that FHR is not always monitored at admission and even less so during the first stage of labor. The reason for the discrepancy between observed and recorded FHRM intervals might be that health workers filled in the partograph after birth to fulfill expectations from administrators as reported in one of the FGDs. This practice was also described in a study in Burkina Faso [23]. Often, women in the latent phase of labor had no record of FHRM because the partograph had not yet been opened. This finding calls for the establishment of an additional document for recording findings of women who are in the latent phase of labor.

In line with findings from another Ugandan study [24], we observed that FHR was not monitored in the second stage of labor. One of the reasons could be that although the WHO guidelines recommend listening to FHR every 5 min during the second stage of labor, the Ugandan clinical guidelines do not give guidance regarding FHRM during this stage [6,17]. The FGDs indicated that monitoring was constrained by the fact that most often births were assisted by one health worker making it difficult to remove sterile gloves in order to perform FHRM in the second stage of labor. While the issue of using sterile gloves in labor is beyond the scope of this paper, it is pertinent to note that midwives did not monitor FHR due to the fear to contaminate sterile gloves. This brings into focus two system issues. First, whether it is necessary to use sterile gloves during delivery. The WHO guidelines and other midwifery literature recommend the use of sterile gloves for delivery and vaginal examinations during labor [20,25]. In addition, the Ugandan guidelines encourage the use of ‘mama kits' which include sterile gloves for use during childbirth [26]. Although evidence shows that clean gloves can be used for vaginal examinations, these studies were among women in labor with intact membranes [27,28]. Secondly, the cost and lack of flexibility when midwives use sterile gloves during the second stage of labor needs to be assessed since in most cases mothers buy their own gloves to be used during childbirth. As it turned out in this study, midwives missed the opportunity to monitor FHR just because they could not ‘take off sterile gloves’ in order to touch the fetoscope. Therefore, there is a need for guidance on how best to manage this uncomfortable situation of either not adhering to clean childbirth or FHRM practices.

Palpation of contractions was not done in any of the observed cases although it was documented in 85.0% of the records. It is important to monitor FHR before and after contractions in order to assess the fetal response to labor stresses [7,29]. We also found that other maternal parameters such as vital signs and urine testing were sub-optimally monitored, which is similar to findings by other studies conducted in Uganda, Malawi and Ethiopia [10,14–16].

The interviews provided possible explanations for the observed sub-optimal FHRM practices. Shortage of staff was mentioned as a key issue and indeed, having an adequate number of staff is a prerequisite to follow FHRM guidelines. Availability of an adequate number of midwives at health facilities has been emphasized by the WHO and other stakeholders in order to reduce maternal and neonatal morbidity and mortality [30,31]. One-to-one care has also been suggested to achieve appropriate FHRM by intermittent auscultation [32]. However, in Uganda and other LMICs, monitoring of FHR as recommended in the guidelines may be far from being realized due to the low staff-patient ratio. Currently, the staffing gap for midwives in Uganda is at 36.0% with one midwife assisting over 350 deliveries a year [33]. This is compounded by the low budget allocation of funds to the health sector which stood at 7.2% in the financial year 2018/2019 [34]. This may affect recruitment of health workers and provision of necessary resources for the care of women in labor [35]. It also raises questions about the appropriateness of adopting international guidelines in such settings as a standard for FHRM. Further, a key issue discussed was lack of fetoscopes, which are the essential low cost tools for FHRM. Obviously, there must be an adequate number of devices available to be able to monitor FHR [30]. A lack of equipment has been described in Uganda before [36] but not having clocks has been rarely documented. Another challenge is inappropriate documentation. This challenge could be mitigated by continuous in-service training to ensure that all staff in the maternity unit monitor and document FHR and other labor parameters uniformly [37,38]. Some of the reasons given for low FHRM in the present study were also described in a study on partograph use in Nigeria and Uganda [36].

Interestingly, we found that women in labor acted as both facilitators of FHRM by reminding the health workers when to be examined and as barriers when they were not present for monitoring. Health providers remarked that women sometimes went back home to perform their daily activities when they were told to ambulate. Involvement of women in their care has been recommended for a positive labor and birth experience [6]. In this setting, there may be a cultural perception of labor as normal and integrated in everyday life; therefore, a woman could still go on with her usual daily activities as she waits to give birth.

Health workers in this study used other measures to check fetal wellbeing. They encouraged the mothers to report reduction in fetal movements which has been shown to be a sign of fetal distress during pregnancy [39]. They also mentioned asking about the color of amniotic fluid; this is one of the parameters that should be recorded on the partograph [6,17].

### Strength and limitations

We believe our three-dimensional scientific approach is a major strength of the present study that provides an in-depth contextual understanding of FHRM in a public hospital in Uganda. Such a comprehensive approach has, to our knowledge, not been used by studies exploring FHRM in LMICs. Monitoring during the second stage of labor has not been investigated previously. The document review and observations were conducted by trained research assistants. With respect to the qualitative data, two of the authors read and initiated the analysis and other authors participated in refining the sub-categories and development of themes. None of the observers, interviewers or authors were involved in the clinical care at this facility, which decreased the risk of bias and increased the possibility of the staff to express themselves freely. A further strength of this study is that our group of authors is multidisciplinary and includes midwives, a neonatologist, pediatricians and an obstetrician.

There were limitations to this study. The study was conducted at one public facility in Northern Uganda which may affect generalizability and transferability to other settings. However, we consider this facility to be a typical example of public hospitals in Uganda. A similar study could be conducted in private health facilities for comparison. The document review and observations were not performed during the exact same time period. We, however, believe that conducting document reviews at two separate time points, and interviews over a long period provided a good understanding of the FHRM practices at this facility in general.

## Conclusions

The actual practice of fetal heart rate monitoring in the rural public hospital in Northern Uganda is sub-optimal in comparison to the existing national and international guidelines. One in four women was not monitored during the first stage of labor and virtually no monitoring was done during the second stage of labor. Some of the barriers to appropriate FHRM include shortage of staff and devices, institutional and documentation challenges and mothers’ unawareness of the importance of monitoring. There is now a documented need for allocation of more health workers and essential resources to the labor ward to enable adequate monitoring of FHR and provision of quality intrapartum care. The working environment for maternity care providers should be improved including the ability to urgently response to fetal emergencies when detected. More so, pregnant women need to be sensitized on the importance of FHRM.

## Data Availability

Data is available on reasonable request from the corresponding author.

## Paper context

Fetal heart rate monitoring is needed for informed decision-making during the intrapartum period to ensure good outcomes. This study uses a mixed methods approach to describe the practice of FHRM. There is sub-optimal monitoring of the FHR during labor with discrepancies in the recorded versus observed practices. Barriers to FHRM include institutional challenges, health worker constraints and mother’s knowledge gaps. This highlights the need to allocate more health workers and resources for FHRM and response.

## Appendix A.

**Table:** Characteristics of women whose records were reviewed

	Records (n = 251)	
	Frequency (%)	Mean ± SD
**Mother’s age in years**		23.3 ± 5.4
< 20	78 (31.1)	
≥20	173 (68.9)	
**Gestational age in weeks**		37.5 ± 2.9
<37	30 (14.5)	
≥37	177 (85.5)	
Missing	44	
**Gravidity**		2.5 ± 1.8
Primigravida	98 (39.2)	
Multigravida	152 (60.8)	
**Cervical dilatation at admission**		5.5 ± 2.2
0–3 cm	39 (16.4)	
4–10 cm	180 (75.6)	
Second stage	19 (8.0)	
Missing	13	
**Shift of admission**		
Day (08:00–14:59 hours)	72 (30.5)	
Evening (15:00–19:59 hours)	48 (20.3)	
Night (20:00–07:59 hours)	116 (49.2)	
Missing	15

**Appendix B.** Outcomes of births from the reviewed records

	Women = 251	
	Frequency (%)	Mean ± SD
**Newborn characteristics**		
**Sex**		
Male	120 (50.6)	
Female	117 (49.4)	
Missing	14	
**Birth weight in grams**		3078 ± 512
< 2500	14 (6.3)	
≥2500	210 (93.7)	
Missing	27	
**Apgar scores at 5 minutes**		9.5 ± 1.9
Score 0 (Stillbirth)	7 (2.8)	
Scores 1–6	8 (3.2)	
Scores ≥7	219 (87.3)	
Missing	17

**Appendix C.** Demographic characteristics of health workers included in the In-depth interviews and FGDs

Study ID	Age	Sex	Qualification	Time since last training in labor monitoring	Clinical practice experience (yrs)	Experience in Maternity care (yrs)
IDI#1	24	F	Enrolled Midwife	none	3	3 weeks
IDI#2	39	M	Obstetrician	6 months	11	4
IDI#3	45	M	Medical Doctor	6 months	2	1.6
IDI#4	30	M	Medical Doctor	1 week	1	2 months
IDI#5	48	F	Diploma Midwife	2 months	17	17
IDI#6	36	F	Diploma Midwife	2 years	11	8
IDI#7	27	F	Diploma Midwife	6 months	4	2
IDI#8	25	M	Medical Doctor	6 months	1	6.5 months
IDI#9	23	F	Enrolled Midwife	2 months	1.5	1.5
IDI#11	30	M	Medical Doctor	2 years	3	2
IDI#10	-	F	Diploma Midwife	5 years	9	7
FGD1M1	43	F	Certificate Midwife	5 months	16	7
FGD1M2	27	F	Certificate Midwife	none	6	4
FGD1M3	35	F	Diploma Midwife	6 years	11	11
FGD1M4	34	F	Diploma Midwife	10 months	12	9
FGD2M1	27	F	Enrolled Midwife	4 years	4	7 months
FGD2M2	39	F	Diploma Midwife	2 years	11	4
FGD3M3	28	F	Enrolled Midwife	4 years	4	1.5

FGD1M1: means Focus group discussion 1 midwife 1.

## References

[1] Fottrell E, Osrin D, Alcock G, et al. Cause-specific neonatal mortality: analysis of 3772 neonatal deaths in Nepal, Bangladesh, Malawi and India. Arch Dis Child Fetal Neonatal Ed. 2015;100:F439. 25972443 10.1136/archdischild-2014-307636 PMC4552925

[2] Uganda Bureau of Statistics(UBOS), ICF. Uganda demographic and health survey 2016. Kampala, Uganda and Rockville, Maryland: UBOS and ICF; 2018.

[3] Ministry of Health. “Why did they die?” Reviewing the evidence to save tomorrow’s mothers and babies. Kmapala, Uganda: Ministry of Health; 2014.

[4] Lawn JE, Blencowe H, Oza S, et al. Every newborn: progress, priorities, and potential beyond survival. Lancet. 2014;384:189–11. 24853593 10.1016/S0140-6736(14)60496-7

[5] Lawn JE, Blencowe H, Waiswa P, et al. Stillbirths: rates, risk factors, and acceleration towards 2030. Lancet. 2016;387:587–603. 26794078 10.1016/S0140-6736(15)00837-5

[6] World Health Organization. WHO recommendations: intrapartum care for a positive childbirth experience. Geneva: World Health Organization; 2018. 30070803

[7] Downe S, Lewis D. FIGO consensus guidelines on intrapartum fetal monitoring: intermittent auscultation. Int J Gynecol Obstet. 2015;13:13–24. 10.1016/j.ijgo.2015.06.019 26433400

[8] Housseine N, Punt MC, Browne JL, et al. Strategies for intrapartum foetal surveillance in low- and middle-income countries: a systematic review. PLoS One. 2018;13:e0206295. 30365564 10.1371/journal.pone.0206295 PMC6203373

[9] Nyamtema AS, Urassa DP, Massawe S, et al. Partogram use in the Dar es Salaam perinatal care study. Int J Gynecol Obstet. 2008;100:37–40. 10.1016/j.ijgo.2007.06.049 17900578

[10] Ogwang S, Karyabakabo Z, Rutebemberwa E. Assessment of partogram use during labour in rujumbura health Sub district, Rukungiri district, Uganda. Afr Health Sci. 2009;9(S1):29–34. PMC2890990 20589158

[11] Gans-Lartey F, O’Brien BA, Gyekye FO, et al. The relationship between the use of the partograph and birth outcomes at Korle-Bu teaching hospital. Midwifery. 2013;29. DOI:10.1016/j.midw.2012.03.002 23146139

[12] Ashish K, Wrammert J, Clark RB, et al. Inadequate fetal heart rate monitoring and poor use of partogram associated with intrapartum stillbirth: a case-referent study in Nepal. BMC Pregnancy Childbirth. 2016;16:233. 27542350 10.1186/s12884-016-1034-5 PMC4991085

[13] Opoku BK, Nguah SB. Utilization of the modified WHO partograph in assessing the progress of labour in a metropolitan area in Ghana. Res J Women’s Health. 2015;2. DOI:10.7243/2054-9865-2-2

[14] Mandiwa C, Zamawe C. Documentation of the partograph in assessing the progress of labour by health care providers in Malawi’s South-West zone. Reprod Health. 2017;14:134. PubMed PMID: 29061189. 29061189 10.1186/s12978-017-0401-7 PMC5654066

[15] Yisma E, Dessalegn B, Astatkie A, et al. Completion of the modified World Health Organization (WHO) partograph during labour in public health institutions of Addis Ababa, Ethiopia. Reprod Health. 2013;10:23. PubMed PMID: 23597239. 23597239 10.1186/1742-4755-10-23 PMC3637818

[16] Namwaya Z, Birungi S, Namutebi E, et al. Partograph initiation and completion: a criteria-based audit study in Uganda. Afr J Midwifery Women’s Health. 2017;11:72–76.

[17] Ministry of Health. Uganda clinical guidelines: national guidelines for management of common conditions. Kampala: Ministry of Health; 2016.

[18] Levitt HM, Bamberg M, Creswell JW, et al. Journal article reporting standards for qualitative primary, qualitative meta-analytic, and mixed methods research in psychology: the APA publications and communications board task force report. Am Psychologist. 2018;73:26. 10.1037/amp0000151 29345485

[19] Stickdorn M, Hormess ME, Lawrence A, et al. This is service design doing: applying service design thinking in the real world. Sebastopol, California: O’Reilly Media, Inc.; 2018.

[20] World Health Organization, UNICEF. Pregnancy, childbirth, postpartum and newborn care: a guide for essential practice. 3rd ed. Geneva: World Health Organization; 2015. 26561684

[21] Elo S, Kyngäs H. The qualitative content analysis process. J Adv Nurs. 2008;62:107–115. 18352969 10.1111/j.1365-2648.2007.04569.x

[22] Erlingsson C, Brysiewicz P. A hands-on guide to doing content analysis. Afr J Emerg Med. 2017;7:93–99. Epub 08/21. PubMed PMID: 30456117. 30456117 10.1016/j.afjem.2017.08.001 PMC6234169

[23] Melberg A, Diallo AH, Storeng KT, et al. Policy, paperwork and ‘postographs’: global indicators and maternity care documentation in rural Burkina Faso. Soc Sci Med. 2018;215:28–35. 30205276 10.1016/j.socscimed.2018.09.001

[24] Tann CJ, Nakakeeto M, Willey BA, et al. Perinatal risk factors for neonatal encephalopathy: an unmatched case-control study. Arch Dis Childhood-Fetal Neonatal Ed. 2018;103:F250–F6. 28780500 10.1136/archdischild-2017-312744 PMC5916101

[25] Lowdermilk DL, Perry SE, Cashion MC, et al. Maternity and women’s health care. 10th ed. St. Louis: Elsevier Health Sciences; 2012.

[26] Ministry of Health, WHO. Maama kit: making childbirth clean and safer 2019. Available from: https://www.afro.who.int/sites/default/files/2017-05/Maama%20Kit%20Final%20copy%28edits%29.pdf

[27] Goetzl L, Houston L, Stephenson K, et al. 627: small incremental costs add up: are sterile gloves on labor and delivery justified? Am J Clin Exp Obstet Gynecol. 2012;206:S281–S2.

[28] Anderson DL, Lanham JS, Grogan SP. Does use of sterile gloves by providers during labor and delivery decrease maternal or neonatal morbidity compared with nonsterile gloves? Evidence-Based Pract. 2018;21:E1–E2.

[29] Ayres-de-Campos D, Spong CY, Chandraharan E. FIGO consensus guidelines on intrapartum fetal monitoring: cardiotocography. Int J Gynaecol Obstet. 2015;131:13–24. Epub 2015/10/05. PubMed PMID: 26433401. 26433401 10.1016/j.ijgo.2015.06.020

[30] World Health Organization. Standards for improving quality of maternal and newborn care in health facilities. Geneva: World Health Organization; 2016. p. 9241511214.

[31] Fistula Care. Improving partograph use in Uganda through coaching and mentoring. New York: Fistula Care/EngenderHealth; 2013. Available from: https://fistulacare.org/archive/files/2/2.3/Uganda_Partograph_technical_brief.pdf

[32] Kripke CC. Why are we using electronic fetal monitoring? Am Fam Physician. 1999;59:2416, 21–2. Epub 1999/ 05/14.PubMed PMID: 10323350. 10323350

[33] UNFPA. Population matters: midwifery services in Uganda Kampala. United Nations Population Fund; 2017 [cited 2019 115]. Available from: https://uganda.unfpa.org/sites/default/files/pub-pdf/Issue%20Brief%202%20-%20Midwifery%20Final.pdf

[34] Ministry of Health of Uganda. Annual health sector performance report: financial year 2018/2019. Kampala, Uganda: Ministry of Health; 2019.

[35] Koblinsky M, Moyer CA, Calvert C, et al. Quality maternity care for every woman, everywhere: a call to action. Lancet. 2016;388:2307–2320. 27642018 10.1016/S0140-6736(16)31333-2

[36] Yang F, Bohren MA, Kyaddondo D, et al. Healthcare providers’ perspectives on labor monitoring in Nigeria and Uganda: a qualitative study on challenges and opportunities. Int J Gynecol Obstet. 2017;139:17–26. 10.1002/ijgo.12379 29218726

[37] Okusanya B, Ogunjimi O, Osanyin G, et al. Effect of training on the knowledge and use of the partograph for low risk pregnancies among health workers in a tertiary hospital in Lagos State, Nigeria. J Community Med Primary Health Care. 2018;30:47–54.

[38] Mezmur H, Semahegn A, Tegegne BS. Health professional’s knowledge and use of the partograph in public health institutions in eastern Ethiopia: a cross-sectional study. BMC Pregnancy Childbirth. 2017;17:291. PubMed PMID: 28877674. 28877674 10.1186/s12884-017-1477-3 PMC5588598

[39] Heazell AEP, Warland J, Stacey T, et al. Stillbirth is associated with perceived alterations in fetal activity - findings from an international case control study. BMC Pregnancy Childbirth. 2017;17:369. PubMed PMID: 29132322. 29132322 10.1186/s12884-017-1555-6 PMC5683455

